# Quantification of kratom alkaloids mitragynine, 7-hydroxy mitragynine, mitragynine pseudoindoxyl, and dihydro-7-hydroxy mitragynine in forensic casework and medicolegal death investigations

**DOI:** 10.1093/jat/bkag090

**Published:** 2026-10-15

**Authors:** Lauren K Eccarius, Sara E Walton, Donna M Papsun, Barry K Logan, Alex J Krotulski

**Affiliations:** Center for Forensic Science Research and Education, Fredric Rieders Family Foundation, Horsham, PA 19044, United States; Center for Forensic Science Research and Education, Fredric Rieders Family Foundation, Horsham, PA 19044, United States; NMS Labs, Horsham, PA 19044, United States; Center for Forensic Science Research and Education, Fredric Rieders Family Foundation, Horsham, PA 19044, United States; NMS Labs, Horsham, PA 19044, United States; Center for Forensic Science Research and Education, Fredric Rieders Family Foundation, Horsham, PA 19044, United States

## Abstract

Mitragynine (MG) is the primary psychoactive component of the kratom plant (*Mitragyna speciosa*). 7-Hydroxy mitragynine (7OH-MG) is found naturally in the plant but in small amounts (<2%). MG is metabolized to 7OH-MG and further to mitragynine pseudoindoxyl (MP), with the latter documented via *in vitro* enzymatic activity. Lack of scheduling and oversight has caused unregulated markets to proliferate with gas stations, smoke shops, and online retailers selling semi-synthetic formulations of these alkaloids under the umbrella term “kratom,” though distinct from botanical kratom. Products containing 7OH-MG, MP, and synthetically derived dihydro-7-hydroxy mitragynine (DH-7OH-MG, aka MGM-15) exhibit greater potencies and activities at the µ-opioid receptor compared to MG and are now highly implicated in intoxications and deaths. These alkaloids present complex analytical and interpretive challenges due to their isomeric forms, incompatibility with certain extraction and instrumental techniques promoting analyte interconversion, instability in blood, and lack of published concentration ranges. A quantitative high-resolution mass spectrometry method for MG, 7OH-MG, MP, and DH-7OH-MG, which prevents interconversion and separates isomers, is presented herein along with stability data and concentrations from more than 200 authentic cases. MG, MP, and DH-7OH-MG were relatively stable in blood, whereas 7OH-MG decreased quickly and by 89% over 112 days with a concurrent increase in MP. In a case subset (*n* = 139), mean ± SD concentrations in blood were 146 ± 477 ng/ml for 7OH-MG (median: 58 ng/ml, range: 0–5000 ng/ml) and 194 ± 239 ng/ml for MP (median: 120 ng/ml, range: 4.8–1900 ng/ml). In a second subset (*n* = 17), the mean ± SD DH-7OH-MG (MGM-15) concentration was 324 ± 669 ng/ml (median: 170 ng/ml, range: 50–2900 ng/ml). Testing for kratom alkaloids is currently extremely limited and/or subject to potential inaccuracies due to interconversion, leaving adverse events and fatalities under-investigated. Continued vigilance is needed as the kratom alkaloid product landscape rapidly evolves.

## Introduction

### Background

The kratom plant (*Mitragyna speciosa*) has been used for centuries in traditional socioreligious ceremonies, for enhancement of worker productivity, and for the treatment of opiate dependence, as well as other medical conditions and common ailments across Southeast Asia. Leaves are typically chewed, brewed into tea, or smoked [1, 2]. Kratom usage in Western countries is more recent, having only gained popularity within the last two decades. Recreational use of kratom is common due to its euphoric and mood-enhancing effects, but it has also been regarded by users as an opioid alternative for the treatment of chronic pain or opioid addiction and withdrawal [2]. Product formulations have gradually shifted from natural kratom to preparations adulterated or enhanced with alkaloids more potent and active than mitragynine (MG) (Fig. 1), the primary psychoactive component of the kratom plant. 7-Hydroxy mitragynine (7OH-MG) (Fig. 1) is a structurally similar alkaloid found naturally in the plant in smaller amounts (<2%) and as an oxidized metabolite of MG. The substitution of this hydroxyl group significantly changes the pharmacological profile of the molecule, increasing the binding affinity of 7OH-MG at the µ-opioid receptor (MOR) and elevating its potency at least 10-fold higher than MG, thereby also enhancing respiratory depressant effects [3–5]. Further metabolic conversion to a more potent substance with opioid activity, mitragynine pseudoindoxyl (MP) (Fig. 1), occurs via enzymatic activity in human plasma. This oxidative rearrangement results in full agonistic activity at the MOR and a 100-fold greater potency than MG [6, 7].

**Figure 1:**
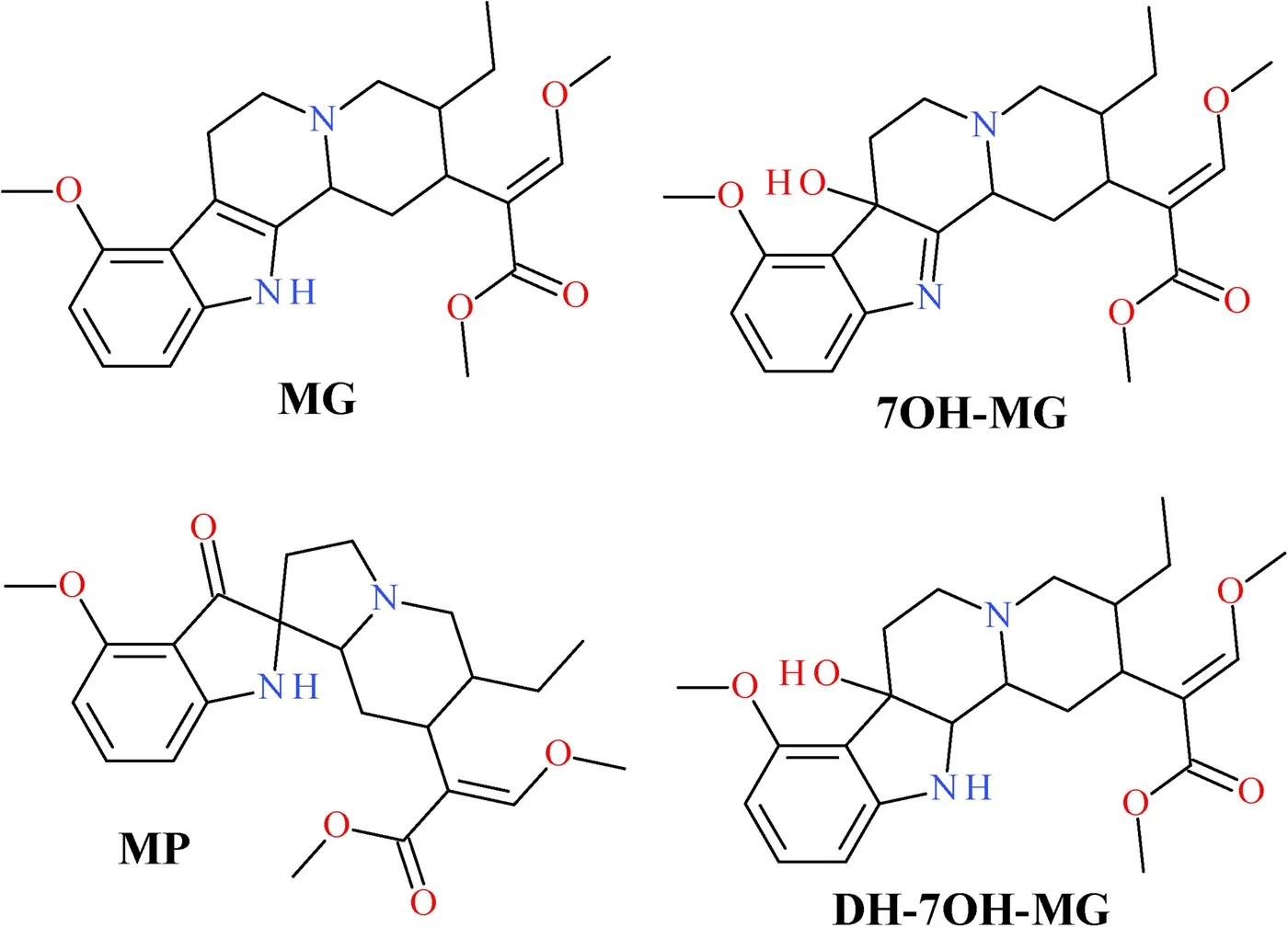
Chemical structures of MG, 7OH-MG, MP, and DH-7OH-MG.

Early suspicions of products with artificially elevated concentrations of 7OH-MG were eventually confirmed with improved testing of commercial “kratom” products. In 2016, Lydecker *et al.* [8] identified multiple products with amounts of 7OH-MG exceeding those naturally found in plant material by up to 500%. This coincided with an attempt by the U.S. Drug Enforcement Administration (DEA) to schedule MG and 7OH-MG, which was ultimately rescinded due to a substantial negative public response, further supported by kratom industry lobbying efforts [9]. As a result, 7OH-MG, followed by other kratom alkaloids, eventually proliferated in unregulated markets with gas stations, smoke shops, and online retailers manufacturing and/or selling “kratom” products in easily accessible and widely available manners.

Semi-synthetic products can appear in numerous forms (e.g. tablets, concentrates, edibles, and beverages) containing 7OH-MG and MP, and these forms have shown increased prevalence in recent years [10–12]. This has posed significant public health and safety concerns, as these products are often marketed as “kratom” with little to no dosage information, while the general public assumption is that the product has prototypical MG formulations [9, 11, 13]. In July 2025, the U.S. Food and Drug Administration (FDA) released a report highlighting the dangers of 7OH-MG and its distinction from natural kratom leaf products. The FDA highlighted various scientific data, including exposure cases from the National Poison Data System, to communicate its concerns, ultimately recommending scheduling of 7OH-MG under the Controlled Substances Act (CSA) [14]. In the absence of further federal action at the time, multiple states moved to pass their own laws banning or restricting 7OH-MG products through various policy actions [9].

In September 2025, a new synthetic (i.e. not naturally occurring) but also semi-synthetic (i.e. derived from MG) kratom alkaloid, dihydro-7-hydroxy mitragynine (DH-7OH-MG, aka MGM-15) (Fig. 1), was detected in materials being offered for sale, further adding to the diversity of products on the market. DH-7OH-MG is reported to be about 30 times more potent than MG [15, 16]. Like other synthetic novel psychoactive substances (NPS), synthetic kratom alkaloids offer numerous opportunities for structural variations [17]. DH-7OH-MG has previously been mentioned alongside 10-fluoro dihydro-7-hydroxy mitragynine (aka MGM-16) in early research, demonstrating the realm of possibilities for new synthetic alkaloids [18]. DH-7OH-MG has been sold as a “research chemical” online and, more recently, under the guise of “proprietary blend” or “nanotech” labeling, following a trajectory similar to other kratom alkaloid products. Marketing has also switched to use of the acronym “DHM,” shifting from “MGM-15,” the name associated with its first literature source [19].

In July 2026, the DEA filed its intent to temporarily place 7OH-MG (above a specified threshold of 0.050% by dry weight), MP, MGM-15, and MGM-16 into Schedule I of the CSA. This designation indicates the substances are viewed to have a high potential for abuse, no currently accepted medical use in treatment in the United States, and a lack of accepted safety for use under medical supervision. This defining action emphasizes the recent dangers of these alkaloids as the DEA finds that “placement of these substances in schedule I of the CSA is necessary in order to avoid an imminent hazard to the public’s safety” [20, 21]. In October 2026, the DEA updated its notices of intent to schedule, based on recommendations from the U.S. Department of Health and Human Services (HHS), to include 7OH-MG and MP (with MG at seperate or combined thresholds not to exceed 1%, as well as not to exceed 1 mg/g or 1 mg/mL without MG), and MGM-15 and MGM-16 (any amount deemed illegal).

### Forensic relevance

Testing for MG is well established. Despite various interpretive challenges, reports regarding MG intoxications and deaths have been extensively published in the literature over the years along with concentration values. Elevated concentrations of MG (e.g. exceeding 1000 ng/ml in blood) are more frequently associated with fatalities and adverse events [22, 23]. In contrast, very little quantitative toxicological data exists for the more potent alkaloids 7OH-MG and MP, along with no reports to date for novel synthetic alkaloids like DH-7OH-MG. While testing is limited, intoxications and deaths associated with these alkaloids have increased, thereby leaving many of these cases under-investigated and not accurately reported.

Many analytical and interpretive challenges are associated with forensic testing of kratom alkaloids. Over 50 alkaloids have been isolated from the kratom plant, with many of these molecules complex due to their stereogenic centers allowing for multiple isomeric forms, most of which do not have commercially available reference standards [2, 24]. The same properties that allow derivation (e.g. adding acid) make routine testing techniques difficult, with interconversion between alkaloids possible upon addition of heat, acid, or base (e.g. conversion of DH-7OH-MG to MG, MG to 7OH-MG, and 7OH-MG to MP) (Fig. 2). This makes testing of kratom alkaloids largely incompatible with gas chromatography mass spectrometry (GC–MS), which utilizes heat, and routine toxicology extraction procedures, which require basic or acidic buffers. In addition to instrumentation choice and sample preparation considerations, analytical methods must be capable of separating isomers for accurate identification and quantification and to avoid interference [9, 24, 25]. Lastly, concerns of analyte stability exist, particularly for MG and 7OH-MG [6, 22].

**Figure 2:**
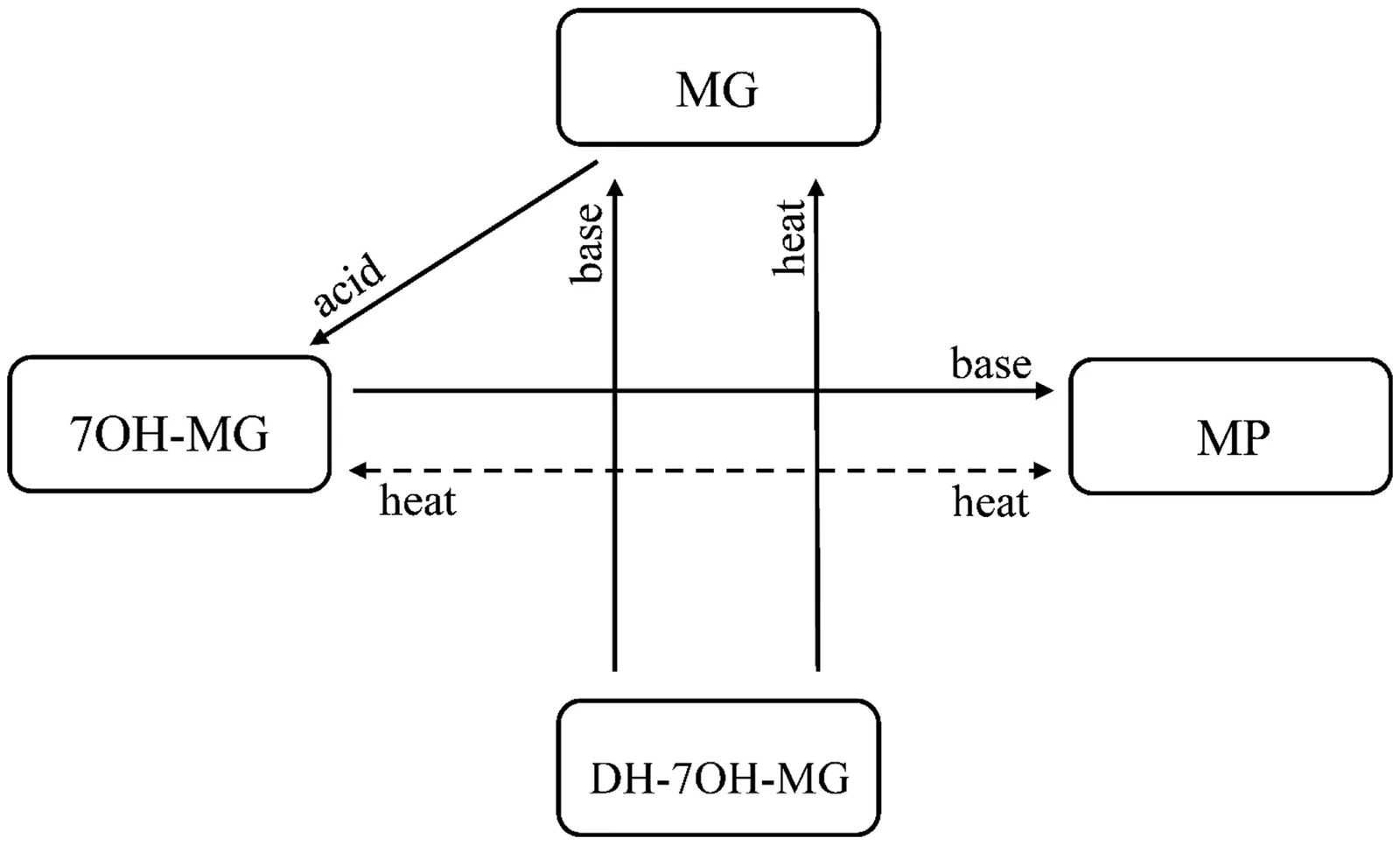
Observed interconversion pathways for kratom alkaloids MG, 7OH-MG, MP, and DH-7OH-MG when applying heat, acid, or base.

This manuscript showcases a quantitative method for MG, 7OH-MG, MP, and DH-7OH-MG in human whole blood (quantitation of other matrices is performed by standard addition and not described herein). The method strategically avoids analyte interconversion through a simple, pH stable, sample preparation technique and allows for chromatographic separation of known isomers including 7OH-MG from MP and MG from its diastereomers (i.e. speciogynine, speciociliatine, and mitraciliatine) (Fig. 3). The method also allows for spectral identification and differentiation from other alkaloids (e.g. paynantheine, corynantheidine, ajmalicine, etc.) through comparison to an in-house library with more than 1200 analytes [26]. A study investigating the stability of MG, 7OH-MG, MP, and DH-7OH-MG in blood was also performed over 12–16 weeks. Ultimately, the quantitative method was used in the analysis of over 200 authentic cases submitted for kratom alkaloid testing from medical examiner and coroner systems, law enforcement agencies, and other forensic laboratories across the USA.

**Figure 3:**
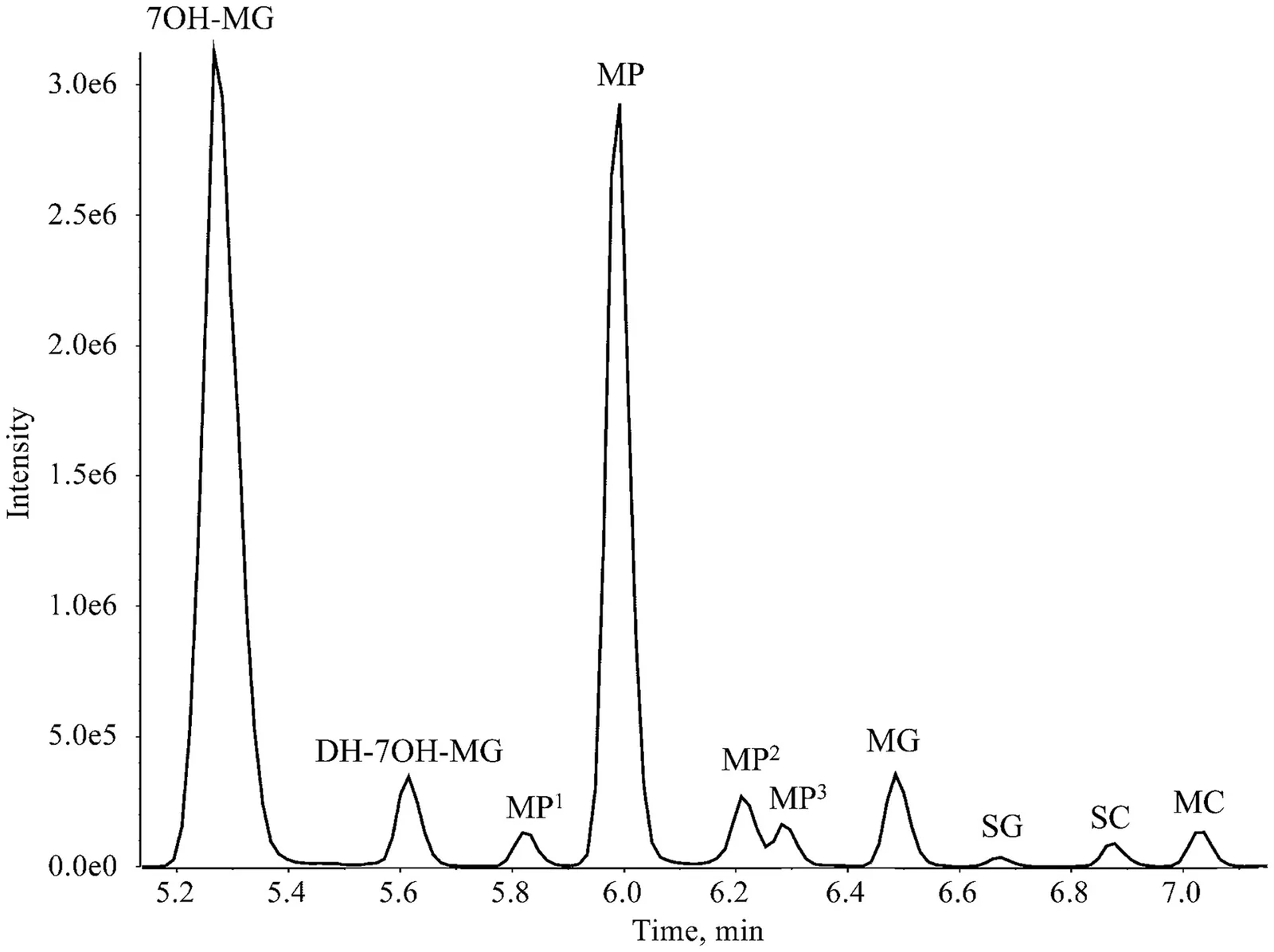
Chromatographic separation of MG, 7OH-MG, MP, DH-7OH-MG, and known MG diastereomers (SG = speciogynine, SC = speciociliatine, MC = mitraciliatine) from an authentic biological specimen. Additional chromatographic peaks labeled as “MP^#^” from hypothesized MP diastereomers due to identical MS/MS spectra; however, confirmation not possible due to unavailable standard reference material.

## Materials and methods

### Materials

Analytical reference standards and certified reference materials (when available) for MG, 7OH-MG, MP, and DH-7OH-MG were purchased from Cayman Chemical Company (Ann Arbor, MI, USA). Fentanyl-D5 (100 µg/ml) was purchased from Cerilliant Corporation (Round Rock, TX, USA). Liquid chromatography mass spectrometry (LC-MS) grade water, methanol, and acetonitrile were purchased from Honeywell—Burdick and Jackson (Muskegon, MI, USA). Ammonium formate was purchased from Sigma-Aldrich (St Louis, MO, USA). Formic acid ampules (1 ml) were purchased from Thermo Fisher Scientific (Waltham, MA, USA). Human whole blood (KOx-NaF) was purchased from BioIVT (Woodbury, NY, USA). BD Vacutainer® Tubes were purchased from BD (Franklin Lakes, NJ, USA).

### Sample preparation

Spiking solutions (final concentrations 0.1, 1.0, and 10 ng/µl) contained all analytes and were prepared by serial dilution in methanol from stock solutions. Calibration curves contained eight non-zero calibrators from 1 to 1000 ng/ml. Quality controls were assessed at 15, 300, and 800 ng/ml. Fentanyl-D5 (100 µl, 0.1 ng/µl) was utilized as the internal standard. Drug-free human whole blood was aliquoted (200 µl) and fortified with appropriate spiking solutions. Authentic biological specimens were also assayed using 200 µl. Stability samples were spiked with each analyte separately at 500 ng/ml and analyzed in triplicate (200 µl aliquots) over 14 or 15 time points across 84 or 112 days alongside freshly prepared calibrators and controls. Stability samples were stored refrigerated (4 °C) between samplings. All samples were extracted by protein precipitation using 600 µl of cold acetonitrile while vortexing. Samples were centrifuged at 4200 rpm for 10 min. One hundred microliter of the organic layer was transferred to autosampler vials with conical inserts, along with 100 µl of initial mobile phase conditions (95:5 A:B). Vials were vortexed prior to analysis via liquid chromatography quadrupole time-of-flight mass spectrometry (LC-QTOF-MS).

### Analytical method

Samples were injected (10 µl) and analyzed on a SCIEX (Framingham, MA, USA) X500R QTOF-MS system coupled with an Exion ultra-high performance liquid chromatograph (UHPLC). Analytical separation was achieved using a Phenomenex® Kinetex C18 column (50 × 3.0 mm, 2.6 µm) maintained at 30 °C. Mobile phases A (10 mM ammonium formate in water, pH 3) and B (0.1% formic acid in 50:50 acetonitrile: methanol) were used in a linear reverse-phase gradient (95A:5B to 5A:95B) with a flow rate of 0.4 ml/min and a total run time of 15.5 min. Acquisition was performed using non-targeted, data-independent (SWATH®) acquisition as previously described by Krotulski *et al.* [26]. Targeted data processing was performed with SCIEX OS software for identification and quantitation of analytes of interest using precursor ions, extracted ion chromatograms, and appropriate fragmentation spectra.

## Results and discussion

### Validation results

The method was validated in accordance with ANSI/ASB 036 Standard Practices for Method Validation in Forensic Toxicology [27]. All evaluated criteria for each analyte were deemed acceptable (Table 1). Limit of detection (LOD) was assessed at 1 ng/ml, and limit of quantitation (LOQ) was assessed at both 1 and 5 ng/ml. The curve fit was determined to be quadratic for all analytes with dynamic quantitative ranges spanning 1 and 1000 ng/ml using a 1/× weighted regression. Correlations (R^2^) were all >0.999, and calibrators and controls were within ±20% of target values. Precision and bias were <15% for all analytes and levels with the exception of MG and DH-7OH-MG at the LOD/LOQ of 1 ng/ml, for which both precision and bias were within 30%. One-to-ten dilutions (100 ng/ml) were deemed acceptable for all analytes. Carryover was not observed after evaluation of the drug-free matrix following injection of the highest calibrator (1000 ng/ml). Recovery, matrix effects, and process efficiency were evaluated, with no significant ion suppression or enhancement observed. Processed samples were stable for up to 96 h.

**Table 1:** Validation results for quantitation of MG, 7OH-MG, MP, and DH-7OH-MG in human whole blood via LC-QTOF-MS.

Validation results	MG	7OH-MG	MP	DH-7OH-MG
Calibration				
Range	1–1000	1–1000	1–1000	1–1000
Model	Quadratic	Quadratic	Quadratic	Quadratic
Weight	1/x	1/x	1/x	1/x
*R* ^2^	0.9991	0.9994	0.9996	0.9994
y-Intercept	1.518	1.322	−0.240	1.304
Bias (%)				
LQC (15)	3.8	4.9	3.7	4.9
MQC (300)	5.2	7.5	1.8	5.4
HQC (800)	8.2	2.9	1.0	3.3
Precision (%)				
LQC (15)	11.1	7.2	9.3	10.1
MQC (300)	9.0	7.2	7.2	9.8
HQC (800)	12.1	6.2	10.6	9.9
LOD/LOQ (1)				
Bias (%)	−4.8	8.5	−14.7	−7.3
Precision (%)	27.3	17.7	18.4	27.3
LOQ (5)				
Bias (%)	5.8	11.1	0.9	0.7
Precision (%)	12.0	14.7	12.9	12.0
Dilution (100)				
Bias (%)	−6.6	−7.0	−5.8	−9.5
Precision (%)	12.4	9.8	10.8	11.8
Recovery (%)	59.4	79.0	78.1	77.7
Matrix effects (%)	131.0	104.5	111.3	126.1
Process efficiency (%)	77.8	82.5	86.9	97.9
Processed sample stability	96 hours	96 hours	96 hours	96 hours

Concentrations are in ng/ml. LQC, low quality control; MQC, mid quality control; HQC, high quality control; LOD, limit of detection; LOQ, limit of quantitation.

### Stability results

Fortified stability samples (preserved human whole blood) were analyzed at 0, 1, 2, 3, 7, 10, 14, 21, 28, 35, 42, 49, 56, and 84 days for all four analytes, and 112 days for 7OH-MG and MP only. MG and DH-7OH-MG did not decrease by more than 20% over 84 days. Over 112 days, MP also remained stable and did not decrease by >10%. 7OH-MG decreased by 89% over 112 days with concurrent increase in MP, demonstrating the conversion of 7OH-MG to MP (Fig. 4). These findings align with previously published literature regarding the stability of MG and 7OH-MG, but are among the first reports for the stability of MP and DH-7OH-MG. MG has previously been reported to be stable for 30 days refrigerated [22, 28]. Osawa *et al.* [28] reported that 7OH-MG was stable for up to 7 days with analyte loss of <20%, and at 60 days, concentrations deviated by over 50%.

**Figure 4:**
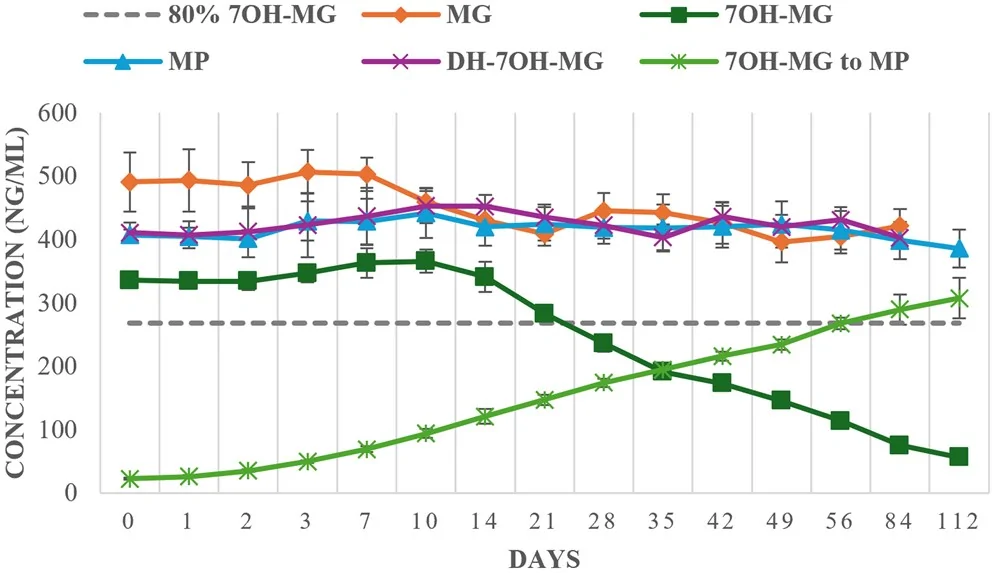
Stability of MG, 7OH-MG, MP, and DH-7OH-MG in preserved human whole blood stored by refrigeration.

The conversion of 7OH-MG to MP and the stability of MP demonstrate the value of MP as a key biomarker in forensic toxicology testing. Testing for MP not only identifies MP cases but also extends the detection window for 7OH-MG cases. However, efforts must still be made for proper storage and expedited testing when 7OH-MG is suspected to ensure the most accurate quantitation and interpretation are achieved for the case.

### Quantitation results—7OH-MG and MP cases

From October 2025 to May 2026, 205 authentic blood samples were analyzed using this quantitative method. A subset of cases (*n* = 139) was identified to exclude those where MG was the suspected primary or co-ingested alkaloid (Table 2). Cases in this subset had a ratio of [MG] to combined [7OH-MG + MP] less than one, categorized as “true” 7OH-MG and/or MP cases. This subset exhibited mean ± SD concentrations of 25 ± 53 ng/ml for MG (median: 9.2 ng/ml, range: 0–430 ng/ml), 146 ± 477 ng/ml for 7OH-MG (median: 58 ng/ml, range: 0–5000 ng/ml), and 194 ± 239 ng/ml for MP (median: 120 ng/ml, range: 4.8–1900 ng/ml). A quantitative value was obtained for MP in every case, further demonstrating its value as a biomarker in 7OH-MG/MP cases and its concerning prevalence as a highly potent alkaloid. Of these cases, the majority were postmortem samples (*n* = 128), with antemortem or hospital admission blood (*n* = 8) and unspecified “blood” (*n* = 4) from various agency types representing a smaller portion of the cases.

**Table 2:** Quantitation results in blood (ng/ml) for characterized 7OH-MG and MP cases.

*n* = 139	MG	7OH-MG	MP
Mean	25 ± 53	146 ± 477	194 ± 239
Median	9.2	58	120
Range	0–430	0–5000	4.8–1900

Distinction between 7OH-MG cases and MP cases is often difficult, with much overlap observed due to enzymatic breakdown, chemical instability, and co-occurrence in popular product formulations. Forensic toxicologists must not only have a viable method that avoids conversion and differentiates 7OH-MG and MP, but other factors must be considered, including time and storage between collection and testing, any products observed at the scene, and drug-use history or any evidence of using certain products. If this information is not available and/or drug products cannot be recovered and/or tested, it is difficult to determine if 7OH-MG, MP, or a combination of the two was ingested based on concentrations alone. The presence of one or both of these potent alkaloids, however, still indicates a potential cause of death or intoxication due to their strong MOR agonism. According to case histories provided, 7OH-MG/MP cases frequently present with symptoms characteristic of opioid overdoses, including frothy secretions around the nose and mouth (known as the “foam cone”), cerebral edema, pulmonary edema and congestion marked by heavy lungs, and urinary retention. These cases also less frequently exhibit co-detections with other illicit drugs or NPS.

### Quantitation results—DH-7OH-MG cases

A second subset (*n* = 17) of the 205 blood samples was identified as “true” DH-7OH-MG cases, where concentrations of DH-7OH-MG, the dominant alkaloid, were greater than 50 ng/ml (Table 3). All were postmortem samples. This subset exhibited a mean ± SD concentration of 95 ± 101 ng/ml for MG (median: 62 ng/ml, range: 3.2–340 ng/ml) and 324 ± 669 ng/ml for DH-7OH-MG (median: 170 ng/ml, range: 50–2900 ng/ml), with 7OH-MG and MP only as minor findings present at much lower or undetectable concentrations. Some of the MG concentrations for these cases do fall within the 100–500 ng/ml range suggested by Papsun *et al.* [22] to be scrutinized as contributory, but they do not approach concentrations exceeding 1000 ng/ml in blood, which are more commonly associated with MG fatalities and adverse events, thereby deemed more likely causative in nature.

**Table 3:** Quantitation results in blood (ng/ml) for characterized DH-7OH-MG cases.

*n* = **17**	MG	7OH-MG	MP	DH-7OH-MG
Mean	95 ± 101	13 ± 15	12 ± 22	324 ± 669
Median	62	9.2	5.2	170
Range	3.2–340	0–50	0–89	50–2900

The observed co-occurrence of MG and DH-7OH-MG in blood is likely due to product formulation, as our laboratory has observed this in drug products as well. DH-7OH-MG exhibits higher potency than MG, so the significant concentrations of DH-7OH-MG in this subset highlight the growing concern of its migration into commercial “kratom” products in addition to its sale on its own. One example of a mixed formulation is the “7 Stax Next Generation Mitragynine Blend” brand of tablets, which is marketed with “proprietary blend” labeling and pseudo-scientific buzzwords like “nano tech mitragynine” with no mention of DH-7OH-MG, despite detection of MG and DH-7OH-MG in the product via LC-QTOF-MS following a test purchase [19]. This same brand of tablets was associated with one of our toxicology cases, found on scene along with a box of wine, prescription drugs, and marijuana gummies as listed in the investigative narrative. In this case, quantitative blood concentrations for the alkaloids were 21 ng/ml (MG), 9.0 ng/ml (7OH-MG), 1.6 ng/ml (MP), and 86 ng/ml (DH-7OH-MG) with ethanol (252 mg/dL), caffeine, cotinine, alprazolam (<10 ng/ml), citalopram (71 ng/ml), naloxone, trazodone (<100 ng/ml), mCPP, and loperamide (<5 ng/ml) as other findings. The cause of death was determined to be the “combined toxic effects of dihydro-7-hydroxy mitragynine, 7-hydroxy mitragynine, mitragynine pseudoindoxyl, mitragynine, and ethanol” with cardiomegaly listed as a contributing cause of death. The manner of death was accident.

## Conclusions

In the evolving landscape of NPS and emerging drugs, high vigilance is required in order to identify changes and trends in drug products that ultimately manifest in forensic toxicology casework and pose various analytical and interpretive challenges. Lack of scheduling and unregulated markets have allowed kratom alkaloid-associated products to flourish and increase in diversity, becoming easily accessible and widely available while use surges across the United States. As a result, significant increases in intoxications and deaths have been observed. With increased potencies compared to MG, testing of kratom alkaloids such as 7OH-MG, MP, and DH-7OH-MG (or others) is imperative for thorough investigation of fatalities, with quantitation serving as an additional aid to interpretation.

This manuscript highlights various considerations for testing these alkaloids, including the evaluation of isomers, analyte interconversion with certain sample preparation and analytical techniques, and the instability of 7OH-MG with subsequent conversion to MP in blood. MP is strongly recommended for inclusion in testing scopes based on its stability and high prevalence in forensic casework. This manuscript also provides some of the first published concentration ranges for these newly emergent alkaloids, but case-by-case interpretation is still required as the observed concentrations range widely, and many additional factors must be considered for distinction of “natural” or botanical kratom and subsequent metabolism versus use of semi-synthetically enhanced products.

Further monitoring in this space is needed as more toxicological data is acquired and the kratom alkaloid product landscape inevitably evolves in response to announcements of recent federal scheduling actions. NPS do not disappear overnight, and analogs may undergo multiple variations while developing strategies to evade legislation before gaining traction as viable replacements in the market. Nimble laboratory testing capabilities, like those described herein, are crucial as these shifts will ultimately manifest in forensic toxicology casework.

## Data Availability

The data underlying this work will be shared upon reasonable request to the corresponding author.
